# Supplementary material for: Weakly supervised regression enables interpretable tumor detection in whole-slide histopathology without negative cases
Source: Sci Rep. 2025 Dec 4;15:43145. doi: 10.1038/s41598-025-27158-8 (PMC12678448; doi:10.1038/s41598-025-27158-8)
Supplement: Supplementary file 1 — Supplementary material 1. [file 41598_2025_27158_MOESM1_ESM.pdf]

Supplementary Material

1 From coarse annotations to tumor percentages

In this section, we demonstrate the methodology used to compute tumor percentages from coarse annotations made by pathologists using pen markers on slides prepared for molecular diagnostics procedures. Below, we provide an example of the Python code used to achieve this. Figure 1 shows two visual examples of this process.

```python
import cv2
import numpy as np

def hex_to_bgr(hex_color):
    """Convert hex color to BGR format for OpenCV usage."""
    hex_color = hex_color.lstrip("#")
    return tuple(int(hex_color[i:i+2], 16) for i in (4, 2, 0))  # Convert to BGR (OpenCV)

def clean_mask(mask, kernel_size=20):
    """Apply morphological operations to clean small holes or noise in a mask."""
    kernel = cv2.getStructuringElement(cv2.MORPH_ELLIPSE, (kernel_size, kernel_size))
    cleaned_mask = cv2.morphologyEx(mask, cv2.MORPH_CLOSE, kernel)  # Fill small holes
    cleaned_mask = cv2.morphologyEx(cleaned_mask, cv2.MORPH_OPEN, kernel)  # Remove noise
    return cleaned_mask

def segment_marker(image, lower_marker, upper_marker):
    """Segment marker annotation in the image using HSV color range for marker."""
    hsv_image = cv2.cvtColor(image, cv2.COLOR_BGR2HSV)
    marker_mask = cv2.inRange(hsv_image, lower_marker, upper_marker)
    contours, _ = cv2.findContours(marker_mask, cv2.RETR_EXTERNAL, cv2.CHAIN_APPROX_SIMPLE)

    filled_marker = np.zeros_like(marker_mask)
    if contours:
        largest_contour = max(contours, key=cv2.contourArea)
        cv2.drawContours(filled_marker, [largest_contour], -1, 255, thickness=cv2.FILLED)

    return clean_mask(filled_marker)

def segment_tissue(image, lower_tissue, upper_tissue):
    """Segment the tissue region in the image using a specified HSV range."""
    hsv_image = cv2.cvtColor(image, cv2.COLOR_BGR2HSV)
    tissue_mask = cv2.inRange(hsv_image, lower_tissue, upper_tissue)
    return clean_mask(tissue_mask)

def calculate_tumor_percentage(tissue_mask, marker_mask):
    """Calculate the percentage of tumor area within the tissue region."""
    tissue_inside_marker = cv2.bitwise_and(tissue_mask, tissue_mask, mask=marker_mask)
    total_tissue_area = np.sum(tissue_mask > 0)
    tissue_in_marker_area = np.sum(tissue_inside_marker > 0)

    if total_tissue_area > 0:
        return (tissue_in_marker_area / total_tissue_area) * 100
    return 0

# Example usage
image_path = '/path/to/image.png'
image = cv2.imread(image_path)

# Define HSV ranges for marker and tissue
lower_marker = np.array([0, 140, 0])   # Adjust for marker color (e.g., blue)
upper_marker = np.array([240, 255, 255])

lower_tissue = np.array([130, 10, 100])  # Adjust for tissue color
upper_tissue = np.array([180, 200, 255])

# Segment tissue and marker areas
marker_mask = segment_marker(image, lower_marker, upper_marker)
tissue_mask = segment_tissue(image, lower_tissue, upper_tissue)

# Calculate tumor percentage
tumor_mask = cv2.bitwise_and(tissue_mask, marker_mask)
tumor_percentage = calculate_tumor_percentage(tissue_mask, marker_mask)
```

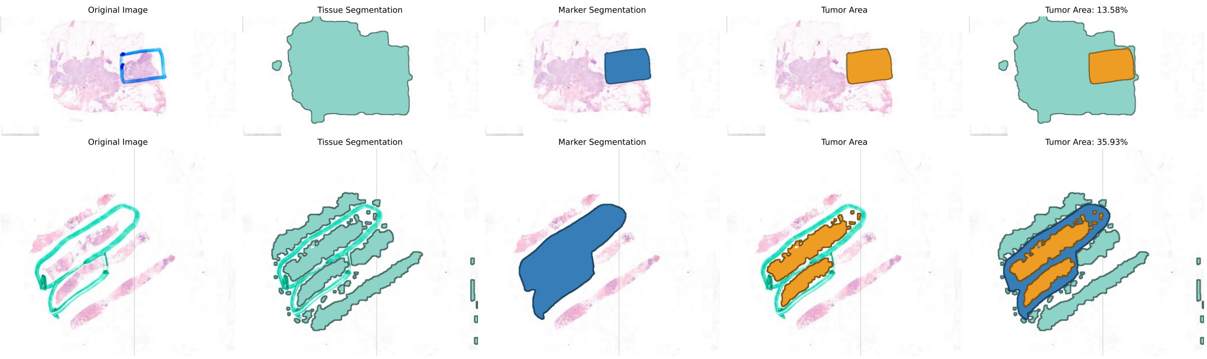

Supplementary Figure 1: Overview of the pipeline for calculating tumor percentages from coarse pathologist annotations.

2 Skin tumor segmentation

Tumor percentages for the COBRA dataset were computed using an in-house skin tumor segmentation algorithm. The model is based on a SeResNeXt50 architecture pretrained on ImageNet and fine-tuned for the task of skin tumor segmentation. The training process was conducted for up to 500 epochs, with early stopping implemented to stop training if performance did not improve on the validation set for 50 consecutive epochs. The dataset used for model development consisted of 319 cases exhaustively annotated, divided into 280 cases for training and 39 cases for validation. Due to the labor-intensive and time-consuming nature of obtaining pixel-level, exhaustive annotations, no separate test set was available for external evaluation. As a result, the model's performance was assessed using the validation set. On the validation set, the model achieved Dice scores of 0.99 for the normal class and 0.95 for the tumor class. The confusion matrix summarizing the performance on the validation set is presented in Figure 2, while Figures 3 and 4 provide qualitative examples of the ground truth masks and the corresponding predicted segmentation masks.

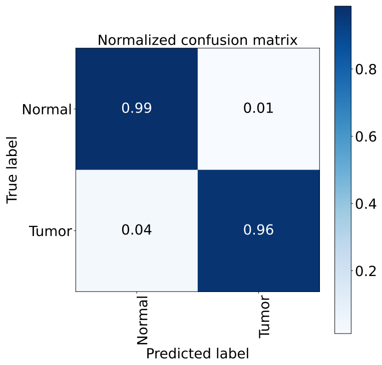

Supplementary Figure 2

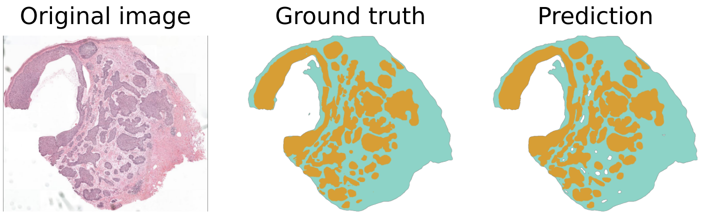

Supplementary Figure 3

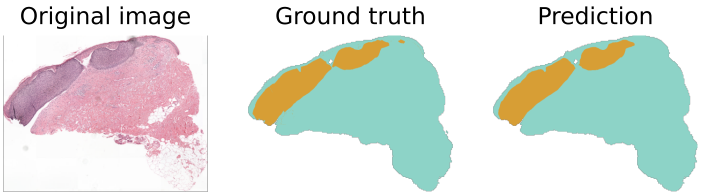

Supplementary Figure 4

Supplementary Figure 5: Overview of model performance: the confusion matrix on the left, and qualitative examples of segmentation results on the right.

3 Regression results

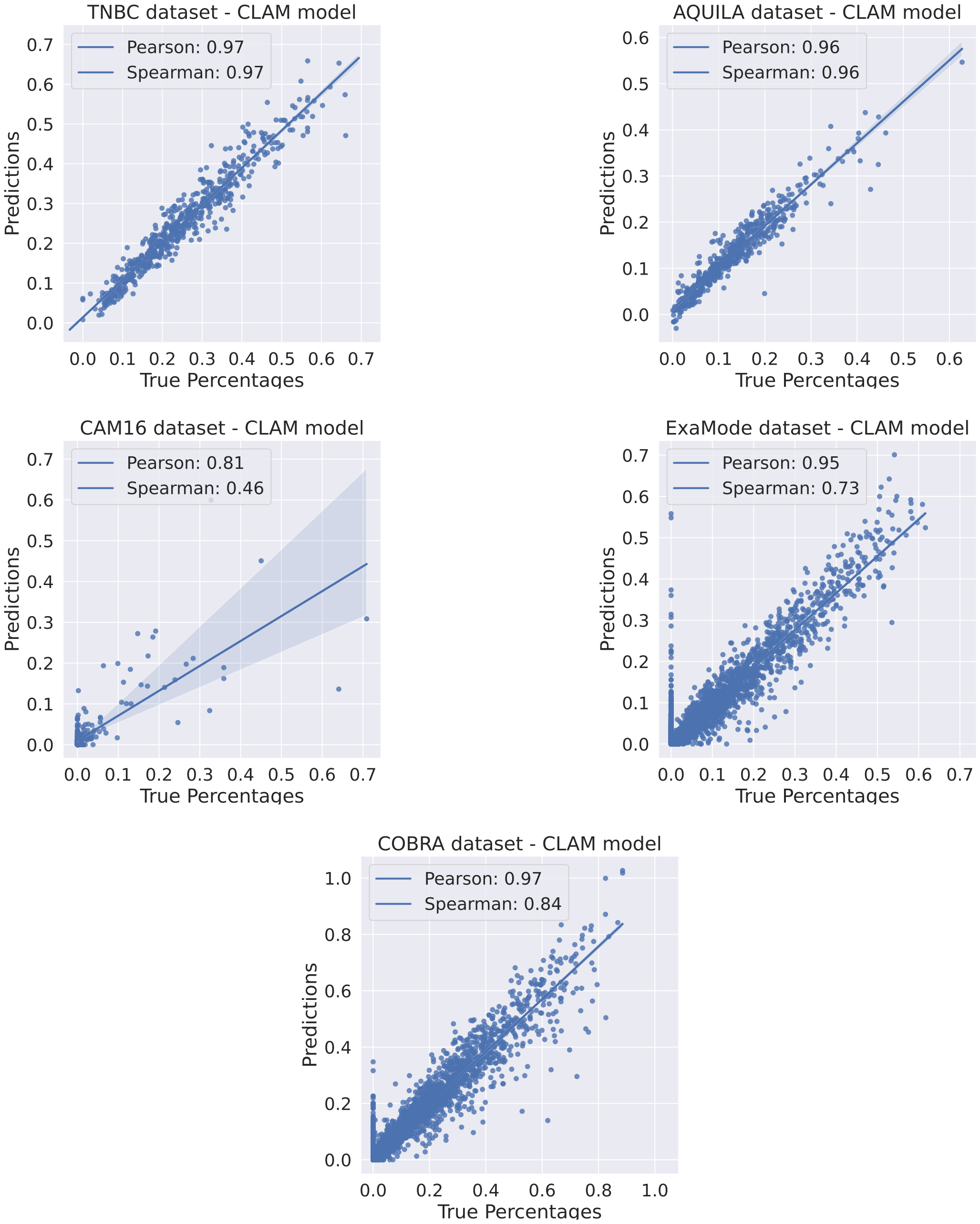

Supplementary Figure 6: Regression plots using CLAM for each dataset showing the relationship between true labels and predicted values.

4 Computational efficiency MIL heads

To assess the computational efficiency of the different MIL heads, we measured per-bag inference latency, throughput, and peak VRAM usage across bag sizes ranging from $N=10^3$–10^5 instances, using identical hardware and settings. For each combination of head type and bag size, a warm-up pass was run to remove initial overhead, followed by 30 repeated forward passes. MeanPool consistently showed the lowest latency and memory footprint, while ABMIL and CLAM were slightly slower and used marginally more VRAM due to the attention mechanism. For all methods, latency increased approximately linearly with N, with the gap between MeanPool and attention-based heads remaining small; throughput trends

Table 1: Latency, throughput, and memory across pooling heads (ResNet50 features, d=1024). Values are measured on bags of size N.

Head	N	Median (ms)	Throughput (bags/s)	Peak VRAM (MB)
MeanPool	1,000	0.3624	2759.6197	18.9663
MeanPool	5,000	0.3779	2646.1312	51.6382
MeanPool	10,000	0.5381	1858.5514	91.1304
MeanPool	20,000	0.9856	1014.6257	169.2554
MeanPool	50,000	2.2486	444.7199	402.7866
MeanPool	100,000	3.6305	275.4442	793.7554
ABMIL	1,000	0.5621	1779.0717	21.5098
ABMIL	5,000	0.5573	1794.4371	55.1377
ABMIL	10,000	0.7104	1407.6848	97.6367
ABMIL	20,000	1.2912	774.4811	180.7588
ABMIL	50,000	2.8833	346.8283	429.2812
ABMIL	100,000	5.4455	183.6363	845.2363
CLAM	1,000	0.6219	1607.9684	21.5142
CLAM	5,000	0.6102	1638.7476	55.1421
CLAM	10,000	0.7102	1408.0798	97.6411
CLAM	20,000	1.2919	774.0719	180.7632
CLAM	50,000	2.9139	343.1880	429.2856
CLAM	100,000	5.4489	183.5225	845.2407

mirrored these differences. Memory usage followed similar trends across methods, with attention adding only a modest constant overhead per instance. In practical terms, these differences are minor compared to the cost of feature extraction, which remains the dominant computational component in WSI MIL pipelines. For this reason, the choice of pooling head is guided primarily by downstream performance and interpretability, with these runtime results provided here for completeness.

5 Ablation on root parameter for amplification technique

To assess the influence of the root exponent in the amplification transformation $\hat{y} = y^{1/n}$, we retrained CLAM with amplification using $n = 3, 5, 7$ on CAM16, COBRA, and ExaMode.

Table 2 reports mean performance over five folds for Pearson and Spearman correlations with the reference tumor percentages, slide-level tumor detection AUC, and interpretability AUCs from attention maps and instance logits.

Across datasets and metrics, performance differences between root settings were generally small, with no consistent best exponent. The fifth root ($n = 5$) provided a good balance between amplifying small tumor values and preserving large ones, and was therefore used as the default in our main experiments.

These results demonstrate that the amplification strategy is robust to the choice of root exponent and can be tuned to the tumor percentage distribution of the target dataset if desired.

6 Inverted Heatmaps Analysis

To better characterize the phenomenon of inverted heatmaps, where regions with low attention scores correspond to tumor regions and vice versa, we quantified the proportion of slides falling into four interpretability categories based on the attention-mask agreement with the ground truth, measured by per–slide AUC:

- **Strong inversion:** $\mathrm{AUC}_{\mathrm{att}} < 0.35$. Attention scores are clearly anti–aligned with tumor regions.
- **Weak / misaligned:** $0.35 \leq \mathrm{AUC}_{\mathrm{att}} < 0.55$. Attention is near–random or mildly anti–aligned.
- **Borderline:** $0.55 \leq \mathrm{AUC}_{\mathrm{att}} < 0.70$. Partial alignment but not reliably localizing tumor.
- **Good:** $\mathrm{AUC}_{\mathrm{att}} \geq 0.70$. Attention maps strongly match tumor regions.

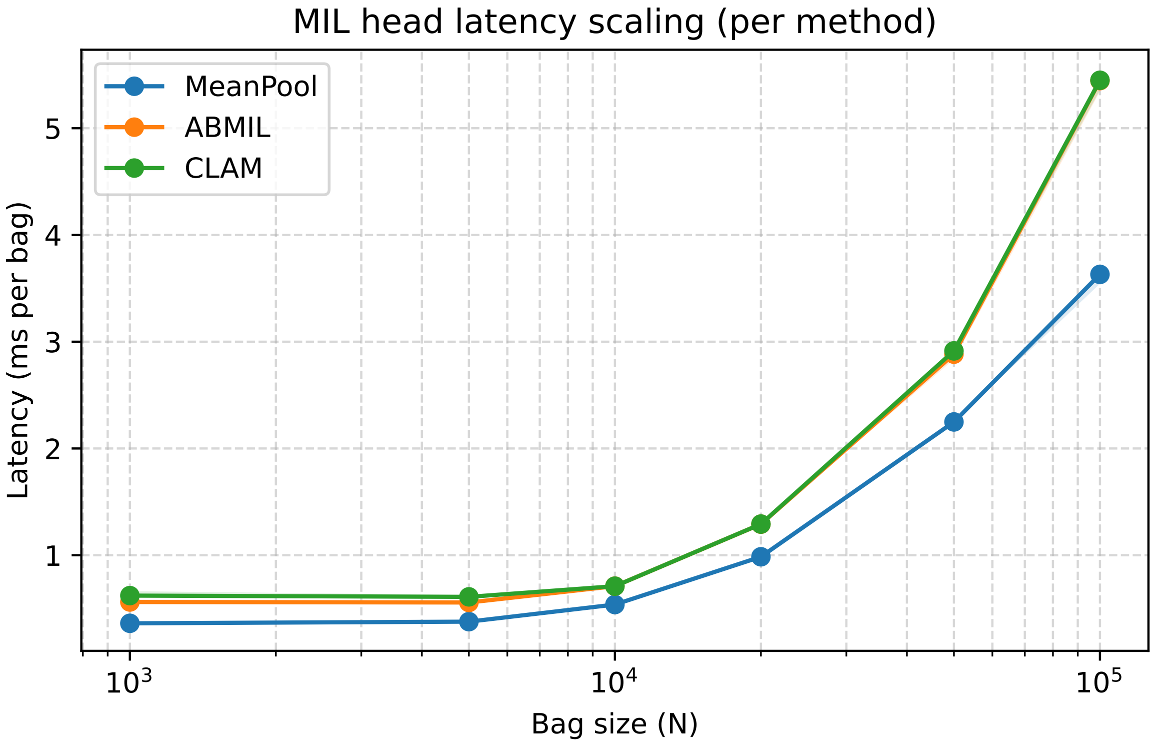

Supplementary Figure 7: Runtime scaling with bag size N for MeanPool, ABMIL, and CLAM.

Table 3 reports the results computed per dataset (CAM16, AQUILA, TNBC, ExaMode, COBRA), model (ABMIL, CLAM), and with/without attention amplification.

We also report the *discrepancy rate* which indicates the proportion of slides where AUCinstance ≥ 0.80 while AUCattention < 0.55. This captures cases where the classifier's instance-level predictions are accurate, but the attention maps fail to localize tumor.

The results in Table 3 show that the prevalence of inversion varies substantially by dataset and model. The issue is most pronounced in TNBC, where both ABMIL and CLAM exhibit high rates of strong inversion ($\approx 23\%$) and weak inversion ($\approx 33\%$), combined with very high discrepancy rates (≈ 0.54). This indicates that, although the instance logits accurately reflect tumor regions, the attention mechanism frequently fails to localize them. AQUILA with ABMIL shows a similar pattern, with strong+weak inversion exceeding 40% and a discrepancy rate of 0.41, while CLAM substantially reduces both metrics on the same dataset. In COBRA, ABMIL still produces inversions (strong+weak $\approx 28\%$, discrepancy 0.24), whereas CLAM lowers these values by more than half. In contrast, ExaMode shows generally lower inversion and discrepancy rates across models. Given that this dataset contains "packed" slides aggregating multiple tissue sections from the same block, tumor regions are often sparse and surrounded by large amounts of normal tissue, which may dilute the interpretability signal.

Across datasets, CLAM consistently outperforms ABMIL in reducing inversion rates, supporting the hypothesis that the clustering loss and instance-level constraints help bias attention toward truly discriminative patches. More interestingly, amplification improves both slide-level tumor detection and interpretability, often increasing the proportion of "good" cases while simultaneously lowering discrepancy rates.

We hypothesize that the phenomenon of inverted attention is partly driven by the regression objective itself: when predicting a continuous tumor percentage, the model can leverage both tumor and non-tumor tissue features to approximate the target value. As a result, attention may focus on non-tumor regions if they provide indirect cues about tumor proportion. This effect is especially pronounced in slides with small tumor areas, where tumor patches contribute less to the regression loss. Amplification increases the loss contribution of low-percentage tumor slides, effectively boosting their influence during training and encouraging attention to focus on true tumor regions.

It is worth noting that such mismatches between slide-level predictive accuracy and localization are

Table 2: Average performance across 5 folds for CLAM with amplification using different root exponents ($n = 3, 5, 7$). Values are mean Pearson and Spearman correlations with the reference tumor percentages, and mean AUC for slide-level tumor detection, attention maps, and instance logits.

Dataset	Root	Pearson	Spearman	AUC Slide	AUC Attention	AUC Instance
CAM16	$n = 3$	0.608	**0.761**	**0.907**	0.930	0.897
	$n = 5$	**0.636**	0.740	0.893	0.935	0.897
	$n = 7$	0.634	0.739	0.893	**0.936**	**0.898**
COBRA	$n = 3$	**0.853**	0.919	**0.993**	**0.915**	0.883
	$n = 5$	0.851	**0.928**	0.989	0.848	**0.895**
	$n = 7$	**0.853**	0.920	**0.993**	**0.915**	0.885
ExaMode	$n = 3$	0.887	0.781	0.963	0.744	**0.756**
	$n = 5$	0.887	0.782	**0.964**	**0.749**	0.754
	$n = 7$	**0.894**	**0.817**	0.961	0.739	0.739

not unique to regression tasks. Similar phenomena have been reported in classification settings, for example in [1], where in NSCLC and RCC subtyping, models sometimes classified slides correctly while retrieving non-diagnostic regions, or misclassified slides despite retrieving patches matching pathologist annotations. In such classification settings, the model is optimized to discriminate between subtypes rather than to explicitly detect tumor regions. This parallels our regression findings, where the objective can encourage attention toward contextual features rather than tumor regions.

Dataset	Model	Amplified	Rate Strong	Rate Weak	Rate Borderline	Rate Good	Discrepancy rate
AQUILA	ABMIL	No	0.130	0.281	0.368	0.219	0.409
AQUILA	CLAM	No	0.007	0.112	0.235	0.644	0.118
CAM16	ABMIL	No	0.108	0.108	0.070	0.090	0.176
CAM16	ABMIL	Yes	0.008	0.025	0.025	0.319	0.03
CAM16	CLAM	No	0.060	0.106	0.103	0.108	0.156
CAM16	CLAM	Yes	0.003	0.003	0.003	0.369	0.002
COBRA	ABMIL	No	0.100	0.185	0.130	0.076	0.239
COBRA	ABMIL	Yes	0.004	0.011	0.030	0.446	0.005
COBRA	CLAM	No	0.022	0.102	0.154	0.213	0.097
COBRA	CLAM	Yes	0.004	0.011	0.030	0.445	0.005
ExaMode	ABMIL	No	0.018	0.063	0.128	0.148	0.049
ExaMode	ABMIL	Yes	0.010	0.029	0.070	0.248	0.013
ExaMode	CLAM	No	0.013	0.023	0.042	0.279	0.015
ExaMode	CLAM	Yes	0.009	0.027	0.065	0.256	0.007
TNBC	ABMIL	No	0.217	0.333	0.239	0.212	0.544
TNBC	CLAM	No	0.227	0.326	0.205	0.242	0.546

Table 3: Interpretability rates per dataset and model, with and without amplification.

References

[1] R. J. Chen, T. Ding, M. Y. Lu, et al. Towards a general-purpose foundation model for computational pathology. *Nature Medicine*, 30:850–862, 2024. `https://doi.org/10.1038/s41591-024-02857-3`.
